# Bomapin is a redox-sensitive nuclear serpin that affects responsiveness of myeloid progenitor cells to growth environment

**DOI:** 10.1186/1471-2121-11-30

**Published:** 2010-04-30

**Authors:** Patrycja Przygodzka, Björn Ramstedt, Tobias Tengel, Göran Larsson, Malgorzata Wilczynska

**Affiliations:** 1 Department of Medical Biochemistry and Biophysics, Umeå University, SE-901 87 Umeå, Sweden

## Abstract

**Background:**

Haematopoiesis is a process of formation of mature blood cells from hematopoietic progenitors in bone marrow. Haematopoietic progenitors are stimulated by growth factors and cytokines to proliferate and differentiate, and they die via apoptosis when these factors are depleted. An aberrant response to growth environment may lead to haematological disorders. Bomapin (serpinb10) is a hematopoietic- and myeloid leukaemia-specific protease inhibitor with unknown function.

**Results:**

We found that the majority of naturally expressed bomapin was located in the nucleus. Both the natural and recombinant bomapin had a disulfide bond which linked the only two bomapin cysteines: one located in the CD-loop and the other near the C-terminus. Computer modelling showed that the cysteines are distant in the reduced bomapin, but can easily be disulfide-linked without distortion of the overall bomapin structure. Low-level ectopic expression of bomapin in bomapin-deficient K562 cells resulted in about 90% increased cell proliferation under normal growth conditions. On the other hand, antisense-downregulation of natural bomapin in U937 cells resulted in a decreased cell proliferation. Bomapin C395S mutant, representing the reduced form of the serpin, had no effect on cell proliferation, suggesting that the disulfide bond-linked conformation of bomapin is biologically important. The bomapin-dependent effect was specific for myeloid cells, since ectopic expression of the serpin in HT1080 cells did not change cell proliferation. In contrast to the survival-promoting activity of bomapin in cells cultured under optimal growth conditions, bomapin enhanced cell apoptosis following growth factor withdrawal.

**Conclusions:**

We propose that bomapin is a redox-sensitive nuclear serpin that augments proliferation or apoptosis of leukaemia cells, depending on growth factors availability.

## Background

Mature blood cells arise in bone marrow from a small population of haematopoietic stem cells during the haematopoietic process. Haematopoietic stem cells give rise to erythroid/myeloid and lymphoid precursors which then continuously proliferate and differentiate to adult blood cells [1]. Biological functions of haematopoietic cells are tightly regulated with multiple growth factors and cytokines, and by interactions with other cells in bone marrow compartment. These factors determine the rate of haematopoietic cells renewal, proliferation, differentiation, and apoptosis. A failure to respond to external stimuli regulating these processes within bone marrow micro-environment may lead to haematological disorders [2,3].

The serpins (serine protease inhibitors) form a protein superfamily, with members identified in all organisms [4]. Although designated as protease inhibitors, serpins have also other functions that are not related to their inhibitory activity. All serpins have a common tertiary structure with a dominant β-sheet A supporting a mobile reactive centre loop (RCL) [5]. The P1-P1' peptide bond in the RCL acts as a bait for target proteases. Serpins inhibit their target proteases by a mechanism that involves cleavage of the RCL and its insertion into β-sheet A, with simultaneous translocation of the covalently-bound protease to the opposite pole of the serpin molecule [6-10]. The serpins that have been studied most extensively are extracellular proteins. They control proteases in various patho-physiological processes, including inflammation, coagulation, and angiogenesis [4,11].

The human genome encodes 35 serpins, which are grouped into nine clades (A-I). The clade B includes 13 intracellular serpins [12]. Some of them are cross-class inhibitors, inhibiting both serine and cysteine proteases [13]. Eight of them have a unique inter-helical loop, the so-called CD-loop [12], which is an important functional domain of the serpins [4]. Although the clade B serpins are thought to regulate apoptosis or cell differentiation, their targets remain mostly unknown.

Bomapin (serpinb10) belongs to the clade B of human serpins. It is expressed only in bone marrow, leukocytes of patients with myeloid leukaemia that correspond to myeloid progenitors [14], and promyelocytic leukaemia cell lines (HL60, THP1, and AML-193), but it is not present in terminally differentiated leukocytes [15]. This protein has a 22 amino acid-long CD-loop that contains a nuclear localization signal [16,17]. Bomapin is assumed to have P1-Arg at its reactive centre which is consistent with *in vitro *inhibition of thrombin and trypsin [14]. Bomapin complex with a possible intracellular protease was detected in HeLa cells over-expressing the serpin, but the protease was not identified [17]. It was proposed that bomapin possibly regulates a protease in the early stages of hematopoietic differentiation [15]. However, the roles of bomapin and its intracellular partners still remain unknown.

The main focus of the present studies is to determine the biological role of the haematopoietic-specific bomapin. We show that naturally expressed human bomapin is located in the nucleus, where it exists in an oxidized form with a single disulfide bond. We show also that bomapin has the capacity to influence the fate of myeloid progenitors/leukaemia cells by enhancing their proliferation under optimal growth conditions, or by sensitizing them to apoptosis when the growth conditions are improper. This function of bomapin may be of particular importance because a tight regulation of cell proliferation and apoptosis, and balance between the two processes, are critical for normal haematopoietic process and for preventing haematological diseases.

## Results

### Bomapin has reactive cysteines that form intramolecular disulfide bond

**Figure 1:**
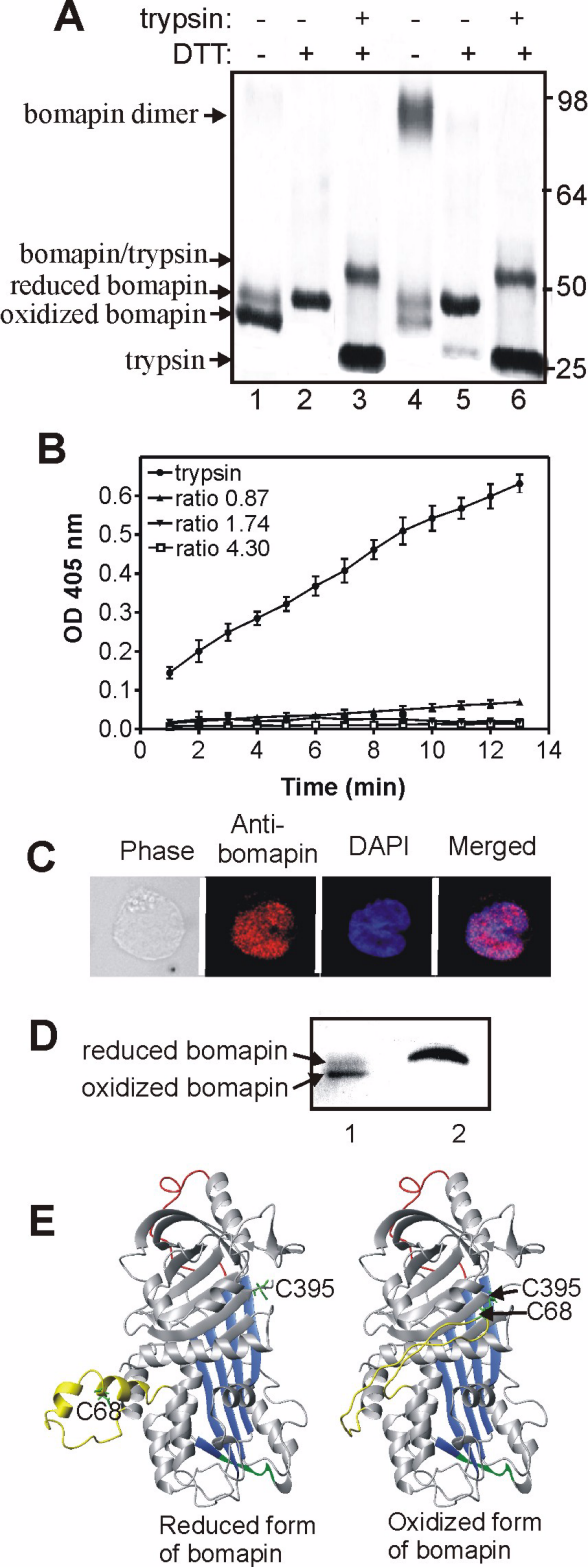
**Conformation of the recombinant and naturally expressed bomapin is redox-dependent**. (A) Purified recombinant bomapin analyzed by SDS-PAGE followed by Coomassie blue staining: lanes 1 and 4, non-reduced bomapin eluted from ion-exchange column in peak 1 and peak 2 (respectively); lanes 2 and 5, DTT-reduced bomapin from peak 1 and peak 2 (respectively); lanes 3 and 6, non-reduced bomapin from peak 1 and peak 2 (respectively) incubated with a 4-fold molar excess of trypsin and then analyzed under reducing conditions. (B) Indirect chromogenic assay to detect inhibitory activity of bomapin. Trypsin alone or trypsin pre-incubated with different amounts of bomapin (at bomapin/trypsin molar ratios as indicated) was mixed with S-2288 chromogenic substrate, and residual trypsin activity was measured at 405 nm in 1 min time intervals. (C) Immuno-localization of naturally expressed bomapin in THP1 cells. The cells were stained with rabbit anti-bomapin antibodies followed by Alexa-Fluor 568-labeled secondary antibody; DNA was stained with DAPI. (D) Bomapin was immunoprecipitated from HL60 cell extract and analyzed by SDS-PAGE followed by western blot: lane 1, non-reduced bomapin; lane 2, DTT-reduced bomapin. (E) *In silico *homology models of bomapin in its reduced and oxidized forms. The central β-sheet A is shown in blue, the reactive centre loop in red, the CD-loop in yellow, and the two cysteine residues in green.

Bomapin was expressed in a thioredoxin reductase deficient *E. coli *strain, and purified by sequential metal-affinity and ion-exchange chromatography. From the ion-exchange column, bomapin eluted in two partially overlapping peaks; the first peak contained bomapin monomer, whereas the second peak contained mainly bomapin dimer (Figure 1A, lanes 1 and 4) and small amount of higher order oligomers (data not shown). Interestingly, the purified monomeric bomapin migrated on non-reducing SDS-PAGE as a strong 40-kDa band, and as a weak 45-kDa band (Figure 1A, lane 1). However, only the 45-kDa band was present under reducing conditions (lane 2). This difference in electrophoretic mobility suggests that the only two bomapin cysteines, C68 (located in the middle of the CD-loop) and C395 (located close to the C-terminus), form an intramolecular disulfide bond. The oxidized and reduced monomeric forms of bomapin, as well as oligomeric species of the protein, were active as inhibitors since they formed an SDS-stable complex with trypsin (Figure 1A, lane 3 and 6). As shown by an indirect chromogenic assay, bomapin was able to inhibit about 90% of trypsin activity at bomapin/trypsin 0.87 molar ratio, and at 1.74 ratio all trypsin was inhibited (Figure 1B). This data suggest that bomapin forms 1:1 complex with trypsin, and support the general model for 1:1 complex formation between serpin and protease [4].

Immunostaining of bomapin in THP1 cells (Figure 1C) and HL-60 cells (data not shown) revealed that naturally expressed bomapin is mainly localized in the nucleus. Since nuclear proteins can be stabilized by disulfide bonds [18,19], the redox status of the nuclear bomapin became of particular interest. Thus, bomapin was immunoprecipitated from HL60 cells and analyzed by 7% SDS-PAGE followed by western blot. The electrophoretic migration of the naturally expressed bomapin (Figure 1D) resembled that of the recombinant protein, suggesting that majority of natural bomapin exists in the oxidized form which contains the intramolecular C68-C395 disulfide bond. In contrast to *E. coli*-expressed bomapin, we have not detected disulfide-linked dimers for the naturally-expressed bomapin.

To provide a structural reference for the redox forms of bomapin, models of the reduced and oxidized forms of bomapin were constructed using homology modelling and simulated annealing calculations (Figure 1E). In the model of reduced bomapin, cysteines C68 and C395 are separated by a distance of about 30 Å, they are surface-exposed and likely to take part in redox reactions. The entire CD-loop (residues 62 to 86) is located on the side of the bomapin molecule. Secondary structure predictions using APSSP2 server http://www.imtech.res.in/raghava/apssp2/ predicts random coils structure from Asn 62 to Glu 72 and between Leu 83 to Ser 86, and a helical tendency between Ser 73 and Asn 82. Thus, the CD-loop can be predicted to be flexible, and it can therefore easily be translocated so that the C68-C395 disulfide bond can be introduced without apparent perturbation of the overall structure of bomapin.

### Wild-type bomapin promotes proliferation of myeloid progenitor cells

**Figure 2:**
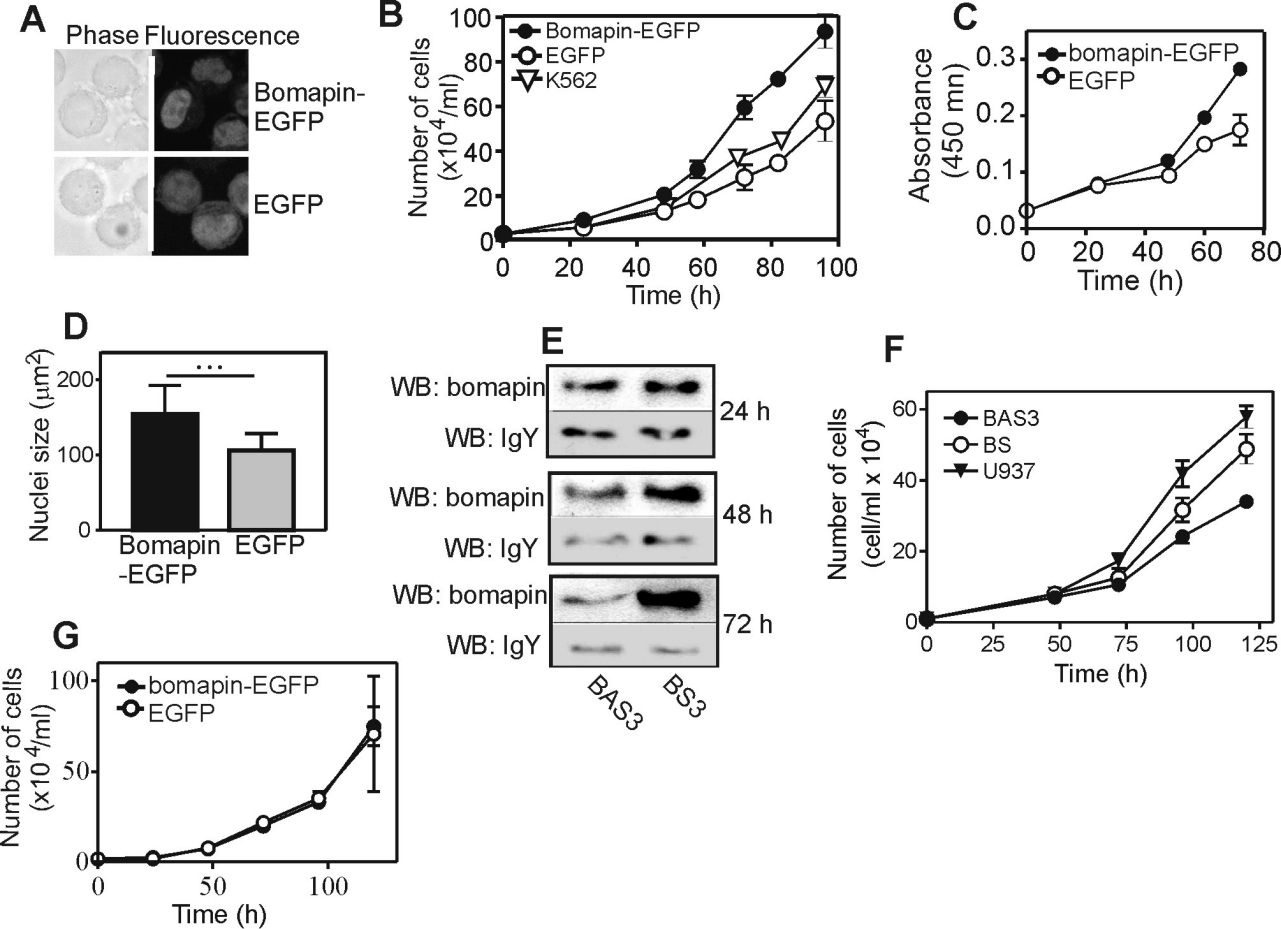
**Wt bomapin promotes proliferation of stably-transfected multi-clonal K562 cells**. (A) Cellular localization of bomapin-EGFP and EGFP in transfected K562 cells. (B) Proliferation of the K562 cells expressing bomapin-EGFP and EGFP, and the wt K562 cells (seeded at 2 × 10^4 ^cell/ml) measured by manual cell counting. The data represent a mean of three independent experiments, each counted in triplicate. (C) Proliferation of the stably transfected K562 cells measured with the WST-1 reagent. (D) Size of nuclei in K562 cells expressing bomapin-EGFP and EGFP. The symbol "..." indicates statistical significance with p < 0.0001 by unpaired t-test. (E) U937 cells were incubated with bomapin-specific antisense (BAS3) and sense (BS) phosphorothioated DNA oligonucleotides (20 nmol/ml); at different time points bomapin was immunoprecipitated with IgY immobilized on NHS-Sepharose and detected with western blot. Western blot of residual amounts of IgY detached from the beads during immunoprecipitation is shown as loading control. (F) U937 cells were seeded at a density of 1 × 10^4 ^cells/ml in the absence or the presence of the antisense BAS3 and corresponding sense BS oligonucleotides, and proliferation was measured by manual counting. The data represent the means of three independent experiments, each counted in triplicate. (G) Proliferation of the HT-1080 cells expressing bomapin-EGFP and EGFP (seeded at 2 × 10^4 ^cell/ml) measured by manual cell counting. The data represent a mean of three independent experiments.

To investigate the role of bomapin, we stably transfected K562 cells with bomapin-EGFP fusion or EGFP alone. As shown in Figure 2A, bomapin-EGFP was localized in the nucleus whereas the control EGFP was distributed in both the nucleus and cytoplasm. The expression level of bomapin-EGFP in K562 cells, measured by bomapin-specific ELISA, was similar to that of native bomapin in THP-1, U937 and HL-60 cells (Table 1).

**Table 1:** Expression levels of natural and recombinant bomapin in cells

Cell line	Bomapin (ng bomapin/mg total protein)
HL-60	0.55 ± 0.02

U937	1.85 ± 0.29

THP-1	2.44 ± 0.58

K562 expressing bomapin-EGFP	1.29 ± 0.26

K562 expressing C395S bomapin-EGFP	1.23 ± 0.21

HT1080 expressing bomapin-EGFP	2.58 ± 0.32

Exponentially growing cells were lysed in a lysis buffer containing protease inhibitor cocktail, and bomapin level was quantified by bomapin-specific ELISA (as described in Methods section).

Proliferation of the bomapin-EGFP and EGFP expressing cells was assayed by manual counting, and by using cell proliferation reagent WST1. As shown in Figure 2B, bomapin-EGFP cells had about 90% higher cell density at 96 h of incubation than those expressing EGFP. Proliferation of wild-type (wt) K562 cells, although slightly higher than for EGFP cells, was still significantly lower than for bomapin-EGFP cells. Bomapin-EGFP cells metabolized the WST1 reagent faster than the EGFP cells (Figure 2C). Finally, the nuclei of bomapin-EGFP cells were about 50% larger than the nuclei of EGFP cells (Figure 2D). Doubling time during the linear phase of cell growth was shorter for bomapin-EGFP cells (25.8 ± 0.6 h) than for EGFP cells (35.6 ± 3.0 h) and wt K562 cells (29.5 ± 2.5 h). However, as shown by DNA-flow cytometry analyses, the cell distribution in the G0/G1, S, and G2/M phases for bomapin-EGFP cells (43.9, 32.5, and 19.9%, respectively) was similar to that for EGFP cells (41.7, 32.2, and 21.5%, respectively), suggesting that bomapin had no effect on distribution of cells during the cell cycle. In addition, the difference in percentage of trypan blue-positive cells for exponentially growing bomapin-EGFP cells (2.4 ± 0.2%) and the control EGFP cells (2.9 ± 0.3%) was not statistically significant, suggesting that the increased proliferation of bomapin-EGFP cells can not be explain by a lower apoptosis rate.

To show that native bomapin also has en effect on cell proliferation, we incubated U937 cells with bomapin-specific antisense (BAS3) or sense (BS) DNA oligonucleotides. Proliferation of all oligonucleotide-treated cells was lower than proliferation of untreated U937 cells, which can possibly result from the known slightly toxic effect of the nucleotides on cells. Nevertheless, the antisense-treated cells had decreased bomapin levels (Figure 2E), and had about 40% lower cell proliferation (Figure 2F), compared to the cells incubated with the sense oligonucleotide. In addition, U937 cells incubated with BAS3 metabolized less WST-1 reagent than the control U937 cells incubated with BS (data not shown).

To find out whether bomapin can influence proliferation of other cells, not only myeloid progenitors, we expressed bomapin in HT-1080 cells. In these cells, bomapin-EGFP was also located in the nucleus (data not shown), and was expressed to a similar level as it was in leukaemia cells (Table 1), but it had no effect on cell proliferation (Figure 2G).

### Bomapin mutant lacking disulfide bond has no effect on cell proliferation

**Figure 3:**
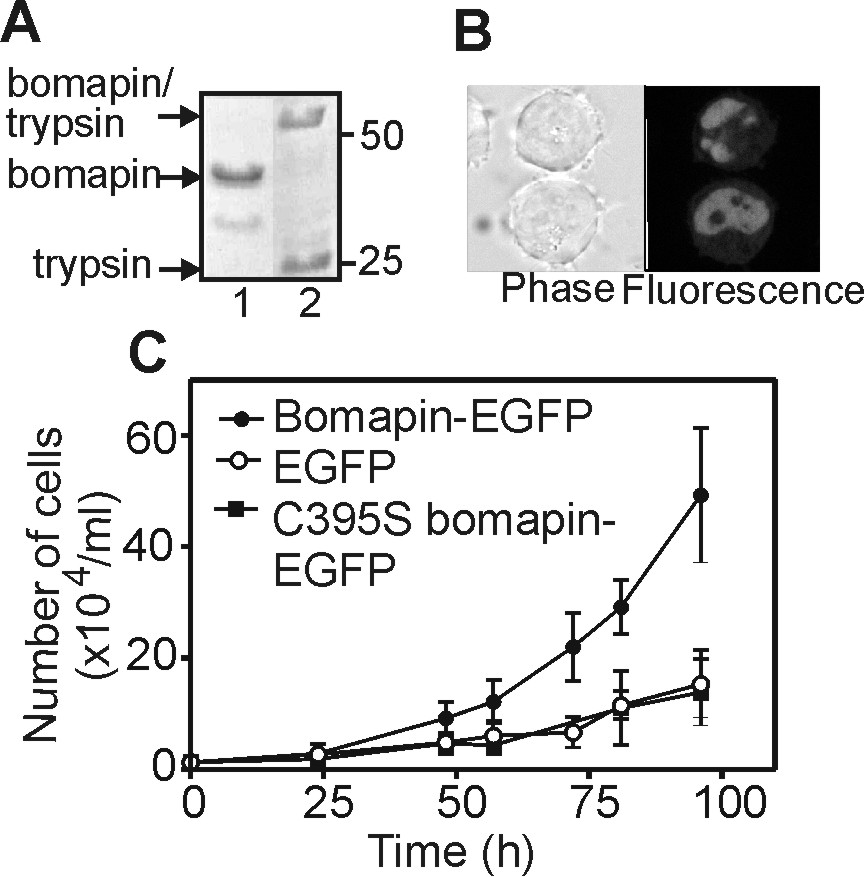
**C395S bomapin mutant, representing reduced form of bomapin, does not enhance proliferation of K562 cells**. (A) SDS-PAGE analysis (followed by Coomassie blue staining) of the E. coli expressed and purified recombinant C395S bomapin mutant (lane 1) and the mutant protein incubated with a 4-fold molar excess of trypsin (lane 2). (B) Cellular localization of C395S-bomapin-EGFP in stably transfected K562 cells. (C) Proliferation of the stably transfected multiclonal K562 cells expressing bomapin-EGFP, C395S-bomapin-EGFP, and EGFP alone, as measured by manual cell counting. Higher proliferation of K562 cells expressing wt bomapin, compared to Fig. 2B, is due to higher generation number of the cells that were used in this experiment.

As majority of natural bomapin was found in the conformation where the CD-loop was linked to C-terminal part of the protein via a disulfide bond, it was of interest whether the oxidized form of bomapin is important for the observed bomapin effect on cell proliferation. Therefore, we created a single-cysteine bomapin mutant (C395S) lacking the disulfide bond, which represents the reduced form of bomapin. Expressed in *E. coli*, this mutant was active as inhibitor and formed an SDS-stable complex with trypsin (Figure 3A). The C395S-bomapin-EGFP fusion expressed in K562 cells had nuclear localization (Figure 3B), as it was shown for wt bomapin (Figure 2A). Expression level of the C395S mutant in K562 cells was also similar to the wild type bomapin (Table 1). However, proliferation of the cells that were expressing the C395S-bomapin-EGFP mutant was identical to that of the control cells expressing EGFP (Figure 3C). This strongly suggests that it is the oxidized form of bomapin that is important for the enhancement of cell proliferation.

### Bomapin enhances cell apoptosis following withdrawal of growth factors

**Figure 4:**
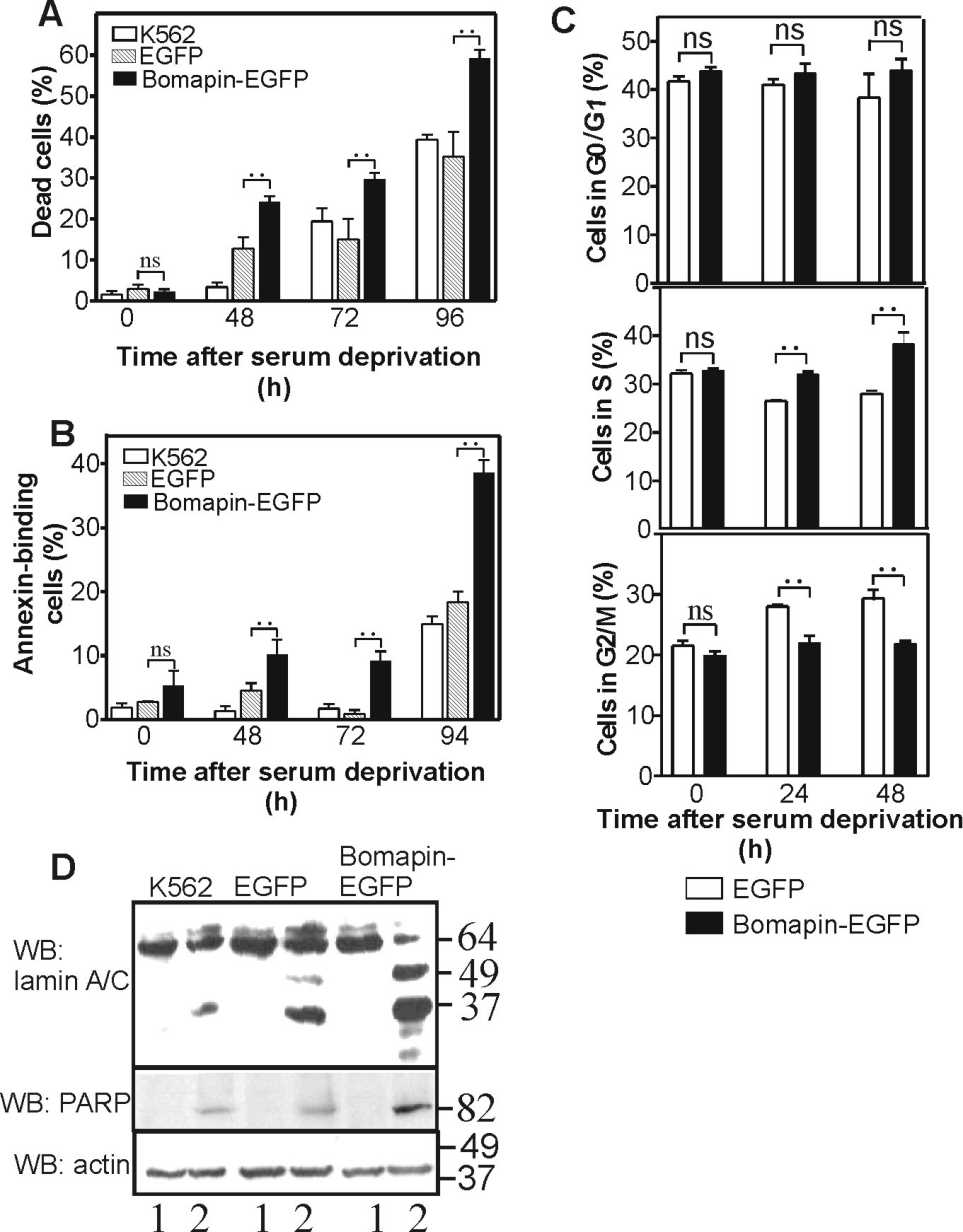
**Bomapin enhances cell apoptosis following growth factors withdrawal**. K562 cells and the cells expressing bomapin-EGFP or EGFP were incubated in the presence or the absence of serum in the media. At different time points, cells were mixed with trypan blue and dead cells were quantified by manual counting (A), or the cells were incubated with annexin-PE-Cys5, and annexin-labelled cells were quantified under fluorescence microscope with excitation and emission wavelengths 488 nm and 670 nm, respectively (B); (C) Progression of cell cycle in bomapin-EGFP and EGFP-expressing K562 cells following serum withdrawal. Percentage of G0/G1, S, and G2/M phases were calculated by deconvolution of DNA content histograms; ns - insignificant; ".." indicates statistical significance with p < 0.05. (D) K562 cells expressing bomapin-EGFP or EGFP were incubated in serum-containing media (lanes 1) or in media without serum (lanes 2) for 48 h. Then, cell extracts were analyzed by western blot with monoclonal antibodies against lamins-A/C and rabbit antibodies against cleaved PARP as apoptotic markers. Western blot for β-actin in the same gel is shown as loading control.

Haematopoietic progenitors deprived of growth factors undergo mitogenic arrest that is followed by apoptosis [20]. Therefore, we tested whether the haematopoietic-specific bomapin has an effect on cell apoptosis. For this purpose, we incubated K562 cells expressing bomapin-EGFP, wt K562 cells, and K562 cells expressing EGFP, under normal growth conditions or in the absence of serum. At different time points of the starvation, dead cells were labelled with trypan blue and counted under microscope. As shown in Figure 4A, the amount of dead cells was about 65-70% higher for bomapin-EGFP cells, than for the parental K562 cells and EGFP cells. The cells were also stained with annexin V-PE-Cys5 and then apoptotic cells showing purple fluorescence on cell membrane were counted under microscope (Figure 4B). Again, there was about 100% more apoptotic cells in the cells expressing bomapin-EGFP than in the control cells. The analysis of cell cycle phases of EGFP-expressing K562 cells cultured without serum showed a progressive increase of cell population in G2/M-phase with a concomitant decrease of cell population in S-phase, compared to the cell distribution under normal growth conditions. Under the same conditions, bomapin-EGFP expression resulted in accumulation of cells in S-phase, while the cell number in G2/M-phase remained constant (Figure 4C).

Apoptosis is characterized by cleavage of several intracellular proteins. Therefore, the cell extracts from EGFP- and bomapin-EGFP-expressing cells were analyzed for two apoptotic markers: lamins-A/C and PARP. There was no, or very little, degradation of lamins-A/C and cleavage of PARP in all the cells under normal growth condition (Figure 4D, lanes 1). However, bomapin-EGFP cells revealed more pronounced degradation of lamins-A/C and higher cleavage of PARP following serum starvation, than the control wt K562 and EGFP-expressing cells (Figure 4D, lanes 2). The data indicate that bomapin expression facilitates apoptosis of K562 cells following withdrawal of growth factors.

## Discussion

Haematopoiesis is the best studied stem cell differentiation process, where hematopoietic progenitors self-renew and differentiate into blood cells, or undergo apoptosis. Any failure to respond to stimuli regulating these processes may lead to leukaemia. Many transcription factors that regulate the haematopoietic processes have been described. However, the downstream pathways, including regulation of various factors by proteases - and control of the latter by specific inhibitors - are less well defined. In this study, we have used leukaemia cells as a model of myeloid progenitor cells. We have shown that the haematopoietic-specific bomapin (serpinb10) is a nuclear, redox-sensitive protein that enhances proliferation of myeloid leukaemia cells under normal growth conditions, and enhances apoptosis of the cells following growth factors withdrawal.

Bomapin has two cysteines: C68 which is located in the long CD-loop, and C395 located close to the C-terminus. Molecular modelling of bomapin suggests that these cysteines in the reduced form of bomapin are distant, solvent-exposed and predicted to be highly reactive. Due to a high potential flexibility/mobility of the CD-loop, the cysteines can be disulfide-linked to form intramolecular disulfide bond without major perturbations of the bomapin structure (Figure 1E). The high reactivity of both cysteines is supported by the fact that bomapin expressed in *E. coli *was present in the form of oxidized monomer (having the two cysteines connected by disulfide bond), and disulfide-linked dimers (Figure 1A). SDS-PAGE analysis of immunoprecipitated naturally expressed bomapin showed that majority of the protein existed in the oxidized monomeric form with intramolecular disulfide bond (Figure 1D). There is a theoretical possibility that bomapin was artificially oxidized during immunoprecipitation and SDS-PAGE, however, the fact that disulfide-linked conformation of bomapin is important for an enhanced cell proliferation (Figure 3C) strongly suggests that the oxidized monomers of bomapin existed already in intact cells. In contrast to the bacterial-expressed bomapin, we have not observed any oligomeric species for the naturally expressed bomapin in leukaemia cells. This could be due to low bomapin levels (Table 1) and crowding effect in nucleus, or known differences in redox environment between nucleus of human cells and bacterial cytoplasm.

Although it is generally known that cytosolic compartment of eukaryotic cells has a high reducing potential which prevents the formation of stable disulfide bonds in proteins, relatively less is known about redox potential of nuclear compartment. However, several reports show that nuclear proteins may exist in oxidized forms with disulfide bonds of functional importance. For example, lamins-A/C, which are structural proteins of nuclear envelope, have to be in the form of disulfide-stabilized dimer to be able to bind chromatin DNA [18]; formation of a single intramolecular disulfide bond in transcription factor Yap1 is responsible for redistribution of the protein into nucleus [19]; disulfide-linked form of clusterin is known to accumulate in nuclei of early apoptotic epithelial cells [21]. Therefore, the existence of a disulfide bond in naturally expressed bomapin is consistent with its nuclear localization (Figure 1C). The disulfide bond also appears to be essential for the function of bomapin since only wt/oxidized bomapin, but not the C395S mutant, was capable of enhancing proliferation of K562 cells (Figure 3C). These data suggest that the oxidised conformation of bomapin is important for an interaction of the serpin with another nuclear protein, and that bomapin effect on cell proliferation might be dependent only on the redox status of bomapin. However, with the current knowledge, we can not exclude a scenario where bomapin inhibitory activity is also involved, but it becomes essential only after the oxidized bomapin binds to its nuclear partner. Bomapin is a second, after its closest homologue plasminogen activator inhibitor type 2 (PAI-2, serpinb2), redox-dependent intracellular serpin. For PAI-2, the formation of a single disulfide bond between cysteines located in the CD-loop and at the bottom of the serpin molecule, induces conformational changes which result in spontaneous non-covalent polymerization of the serpin [22,23], but biological function of the conformational switch is unknown.

To study intracellular function of bomapin, we took advantage of the fact that the human K562 cells do not express bomapin naturally (real-time PCR and immunoprecipitation, data not shown; [15]), and stably transfected the cells with bomapin-EGFP fusion, or EGFP as a control. Consistent with previous studies on HeLa cells over-expressing GFP-bomapin [16], the bomapin-EGFP fusion in K562 cells had a dominant nuclear distribution (Figure 2A). Expression of bomapin-EGFP in K562 cells resulted in about 90% higher cell proliferation (Figure 2B and 2C), and a significant shortening of the cell cycle without changes in distribution of cells in different phases of cell cycle. Bomapin-EGFP expressing cells had also bigger nuclei than the control cells (Figure 2D). On the other hand, down regulation of bomapin expression in U937 cells by means of antisense oligonucleotides resulted in a decreased cell proliferation (Figure 2F), suggesting that the bomapin effect on cell proliferation was not specific for the K562 cells only. However, the effect of bomapin on cell proliferation was leukaemia/haematopoietic-specific because expression of bomapin-EGFP in the human fibrosarcoma HT1080 cells did not change proliferation of the cells (Figure 2G). This strongly suggests that bomapin needs a haematopoietic-specific partner protein to enhance cell proliferation. Two other serpins from clade B have been reported to influence cell proliferation. The first one is rat trespin which is believed to be a homolog of human bomapin, but it is expressed in various tissues whereas bomapin is bone marrow-specific [15,24]; over-expression of trespin in human embryonic kidney epithelial cell line (Hek293) resulted in an increased proliferation of the cells [24]. The second one is kidney-specific mouse megsin which is responsible for increased proliferation of messangial cells in megsin-transgenic mice [25]. The mechanism(s) behind serpin-dependent enhancement of cell proliferation remains yet unknown.

Bone marrow haematopoietic progenitors, quiescent without stimulation, can be activated to proliferate and to differentiate by cytokines and growth factors. When growth factor levels decrease, the cells undergo mitotic arrest followed by apoptosis that leads to termination of cell expansion [3,20,26]. In contrast, leukemic cells cultured in the absence of growth factors can continue to proliferate and evade apoptosis for a long time. In the case of K562 cells, the aberrant Bcr/Abl fusion kinase activates both proliferation and anti-apoptotic signals that are responsible for relatively high proliferation rate of these cells, and their resistance to apoptosis [27]. However, bomapin-EGFP expressing K562 cells cultured without serum showed an increased cell accumulation in S-phase and increased apoptosis, compared to the control cells expressing EGFP (Figure 4). Therefore, bomapin antagonise the anti-apoptotic properties of Bcr/Abl fusion and sensitizes K562 cells to apoptosis when growth factors are absent.

## Conclusions

Hematopoiesis requires a tight balance between proliferation and apoptosis of hematopoietic progenitors. This balance is controlled by many factors, including cytokines and growth factors. Although precise signalling pathways and downstream effectors balancing proliferation and apoptosis are not fully known, they may involve AKT, E2F1/Rb protein, and/or Myc signalling pathways [28]. These signalling pathways respond to growth factor levels by inducing cell proliferation or cell apoptosis. Here we show that bomapin, a nuclear and redox-sensitive protein, can stimulate proliferation of myeloid progenitor cells under normal growth condition, and can increase apoptosis of the cells following the growth factors removal. We therefore propose that bomapin is involved in a pathway that sensitizes myeloid progenitor cells to growth environment. Since proper regulation of cellular proliferation and apoptosis is essential for normal hematopoiesis and for prevention of leukemic transformation, the function of bomapin described here may be of considerable importance.

## Methods

### Expression and purification of bomapin from *E. coli*

The *BamH1 *and *Xho1 *restriction sites in the pET15b vector (Novagen) were flipped using Quick Change Site-Directed Mutagenesis Kit (Stratagene) and primers: 5'-CTGGTGCCGCGCGGCAGCGGATCCCTCCTCGAGCCGGCTGCTAACAAAGCCCG-3' and 5'-CGGGCTTTGTTAGCAGCCGGCTCGAGGAGGGATCCGCTGCCGCGCGGCACCAG-3'. Bomapin cDNA was excised from the PGEX-4T-1-bomapin vector (a kind gift from Dr. R Schleef [14]) using *Xho1 *and *BamH1*, and cloned to the "flipped" pET15b vector. The C395S mutation was introduced with primers: 5'-CTTTTTTATGGAAGATTATCCTCC CCCTAA-3' and 5'-TTAGGGGGAGGATAATCTTCCATAAAAAAG-3', and followed by full-length cDNA sequencing. *E. coli *AD494(DE3) (Novagen) was then transformed and induced with 0.1 mM IPTG (isopropyl-β-D-thiogalactoside) to produce histidine-tagged bomapin. Bomapin was purified under native conditions on a TALON column (Clontech Laboratories) according to the manufacturer's instructions. Then, it was desalted on a NAP-25 column, loaded onto a MonoQ HR 5/5 column (both GE Healthcare), and eluted with NaCl gradient in 20 mM HEPES, pH 7.0. The yields of purification of wt bomapin and the C395S mutant were 1.2 mg and 0.13 mg per 1 L of bacterial culture, respectively.

### Assay for inhibitory activity of bomapin

The inhibitory activity of bomapin against bovine trypsin (Sigma) was measured by a chromogenic assay using the substrate S-2488 (Chromogenics). Bomapin and trypsin were diluted in activity assay buffer (0.05 M Tris/HCl, pH 7.5, containing 0.15 M NaCl and 0.05% Tween 80) to final concentrations 15 μg/ml and 10 μg/ml, respectively. Trypsin (10 μl) was mixed in a 96-well plate with various amounts of bomapin to obtain bomapin/trypsin molar ratios 0.87, 1.74, and 4.3 (in total volume 100 μl), and incubated for 5 min at room temperature. Then 100 μl of 0.4 mM S-2488 substrate was added and absorbance at 405 nm was measured at 1 min intervals in a Titertek Multiscan spectrophotometer.

### Cell culture

We have used leukaemia cell lines originating from different subclasses of myeloid leukaemia. However, the common characteristic of these cells is that their differentiation is blocked at early stages of myeloid differentiation: the HL-60 (acute promyelocytic leukaemia) and K562 (chronic myelogenous leukaemia) cells represent multipotent myeloid progenitors, and U937 (histiocytic lymphoma) and THP-1 (acute monocytic leukaemia) cells represent monocytic progenitors. The K562 cells (naturally bomapin-deficient), as well as U937, HL60, and THP1 cells (all expressing bomapin) were cultured in RPMI-1640 medium. The human fibrosarcoma HT-1080 cells (bomapin-deficient) were cultured in DMEM medium. All media were substituted with 10% foetal bovine serum (FBS), 2 mM L-glutamine, streptomycin (100 μg/ml), and penicillin (100 U/ml) (all from Gibco BRL). All the cells were from ECACC.

### Expression of bomapin in eukaryotic cells and proliferation assays

Bomapin cDNA was amplified from the PGEX-4T-1-bomapin vector by PCR using *pfu*-DNA polymerase and primers 5'-AAAGCTGAATTCTCGAGGCACCATGGACTCTCTAGCAACATCAATC-3' and 5'-GGCGACCGGATCCGCGGGGGAGCATAATCTTCCATAAAA-3' containing *XhoI *and *BamH1 *cleavage sites (respectively). The PCR product was cloned into pd2EGFP-N1 vector (Clontech) using the above restriction sites, and fully sequenced. The C395S mutation was introduced with primers: 5'-CTTTTTTATGGAAGATTATCCTCCCCCTAA-3' and 5'-TTAGGGGGAGGATAATCTTCCATAAAA AAG-3', and followed by full-length cDNA sequencing. The pd2EGFP-N1 vector encodes for a destabilized bomapin-EGFP fusion protein with a half-life of about 2 h. K562 cells were transfected using lipofectamine 2000 (Life Technologies), selected with Geneticin (0.6 mg/ml, Life Technologies), and sorted with a fluorescence-activated cell sorter (Becton Dickinson). To correct for clone variation, all experiments were performed on a multiclonal pool of the stably transfected cells. Proliferation of K562 cells expressing wt bomapin was increasing with increasing cell generation number, whereas proliferation of K562 expressing the C395S bomapin mutant was decreasing with higher generation number. Cell proliferation was assayed by manual counting of trypan blue-excluded cells, and with Cell Proliferation Reagent WST-1 (4-[3-(4-Iodophenyl)-2-(4-nitrophenyl)-2H-5-tetrazolio]-1,3-benzene disulfonate; Roche).

### Apoptosis assays

Apoptosis in K562 cells and the K562 cells stably expressing EGFP or bomapin-EGFP was induced by serum starvation. At different time points, the amount of dead cells was quantified by manual cell counting in the presence of trypan blue. For detection of apoptotic cells, 1 × 10^5 ^cells in 20 mM Hepes pH 7.1, supplemented with 0.14 M NaCl and 25 mM CaCl2, were incubated with annexin V-PE-Cys5 (Abcam) according to manufacturer's instruction, and cell-surface annexin-labelled cells were counted under fluorescent microscope (with excitation and emission wavelengths 488 nm and 670 nm, respectively); non-labelled cells were counted under normal phase light. In addition, cell extracts at different times of serum starvation were prepared in 10 mM Tris/HCl, pH 7.5 with 0.1 M NaCl, 1 mM EDTA 1% Triton X-100, and protease inhibitor cocktail (Roche Diagnostic), and analysed by western blot with antibodies for two apoptotic markers: lamins-A/C (rabbit antibodies against C-terminal fragment of Lamin A; Biolegend) and cleaved PARP (rabbit antibodies specific for cleaved PARP; Abcam), and for β-actin (with monoclonal antibodies to human β-actin; Sigma) as loading control.

### Flow cytometry

For cell-cycle analyses, cells were fixed in 70% ethanol, treated with RNase A (500 μg/ml), and stained with propidium iodide (10 μg/ml). Cell cycle analysis was performed on a flow cytometer (Cytomix FC500; Backman Coulter).

### Antibodies against bomapin and ELISA

His-tagged bomapin was used for immunization of a rabbit and chicken. Total IgY were purified from egg yolk (all at AgriSera AB, Sweden). This IgY and rabbit antiserum were depleted from anti-his-tag antibodies on immobilized his-tagged PAI-2 (serpinb2), and bomapin-specific antibodies were purified on bomapin-NHS-Sepharose. These antibodies were used for immunoprecipitation, western blot, and ELISA. For ELISA, IgY (2 μg/ml) were used for coating, and rabbit anti-bomapin antibodies (0.2 μg/ml) as first antibodies; these were followed by anti-rabbit horseradish peroxidase-conjugated secondary antibodies (Promega). The working range for the ELISA was from 1 to 60 ng/ml. For immunostaining, the antibodies were further pre-incubated with proteins from K562 cell extract that were immobilized on NHS-Sepharose.

### Immunoprecipitation

The cells (2-5 × 10^8 ^cells) were lysed in 50 mM HEPES pH 7.4, 150 mM NaCl, 5% glycerol, 2.5 mM EDTA, 1% NP-40 and protease inhibitor cocktail (Roche Diagnostic). The cell lysates were centrifuged, and then immunoprecipitated with anti-bomapin IgY immobilized on NHS-Sepharose. The samples were analyzed by SDS-PAGE followed by western blot. Bomapin was detected using rabbit anti-bomapin antibodies, followed by anti-rabbit horseradish peroxidase-conjugated secondary antibodies (Promega). Immunoreactive bands were visualized with the Enhanced Chemiluminescence Kit (ECL, GE Helthcare).

### Immunofluorescence microscopy of cells

Cells were seeded on poly-lysine coated cover slips (Sigma), fixed in 3% paraformaldehyde, permeabilised with 0.1% Triton X-100, and blocked with 2% goat serum in PBS. Bomapin was stained with rabbit anti-bomapin antibodies, followed by appropriate secondary antibodies conjugated with Alexa Fluor 568 (Molecular Probes). In the case of cells expressing bomapin-EGFP-fusion and EGFP, fluorescence of EGFP was detected. Images were captured with oil immersion using an AxioImager.Z1 microscope with ApoTome, lenses PLAN-APOCHROMAT 63 × 1.4, Axio Cam MRm (all from Zeiss) and Axio Vision software version 4.5. For quantification of the nuclear size, proliferating K562 cells expressing EGFP or bomapin-EGFP were DAPI-stained, the images were captured, and the nuclear areas were measured using the Axio Vision software.

### Antisense oligonucleotides

U937 cells were incubated with antisense (BAS3: 5'-GCCCTATACTTTAAAGGAATC-3') or sense (BS: 5'-GCCCTATACTTTAAAGGAATC-3') phosphorothioated DNA oligonucleotides (DNA Technology A/S, Denmark) at 20 nmol/ml, using standard growth conditions. Cell proliferation was quantified by manual counting and with Cell Proliferation Reagent WST-1. Bomapin expression was assessed by immunoprecipitation from equal amounts of cell extracts (0.7 mg total protein) using anti-bomapin IgY immobilized on NHS-Sepharose. This was followed by western blot with bomapin-specific rabbit antibodies. For loading control, a small amount of IgY that normally detaches from IgY-Sepharose during the immunoprecipitation was detected with rabbit anti-chicken antibodies coupled to alkaline peroxidase (Sigma).

### *In silico *models of reduced and oxidized bomapin

The model of reduced bomapin was generated from the bomapin sequence (P48595) using the SWISS-MODEL server (version 3.5) [29] with the coordinates of PAI-2 (1by7), serpinB1 (1hle), and ovalbumin (1ova) as templates. This model was energy-minimized further and used to calculate the oxidized bomapin structure using parallhdg.pro force field within X-PLOR version 3.851 [30]. To create the C68-C395 disulfide bond, residues between positions 60-87 and 394-397 were allowed to move freely under the simulated annealing [31] and simulated annealing refinement protocols, whereas all other residues were kept in fixed positions to preserve the secondary structure elements and the overall fold of bomapin. The disulfide bond was introduced as a distance constraint (2.02 ± 0.05 Å) between the two sulphur atoms of the cysteines. Altogether, 20 structures were calculated and the structure with the lowest total energy was chosen as being representative of the ensemble of structures. The models of reduced and oxidized bomapin have been deposited to the Protein Model Data Base [32]http://mi.caspur.it/PMDB/ with accession codes PM0074678 and PM0074679, respectively.

### General methods

SDS-PAGE (10%) was performed as described previously [33], and followed by Coomassie Blue staining or immunoblotting [34]. Protein concentration was determined using the bicinchoninic acid assay (Pierce) or the Coomassie Plus Protein Reagent (Bio-Rad), with bovine serum albumin as protein standard.

### Statistical analysis

Data are expressed as mean values ± SEM, and analysed for statistical significance using uncoupled t-student test with confidence 95%; p value of <0.05 was considered as significant.

## Authors' contributions

PP carried cell culture, designed and performed proliferation and apoptosis assays. BR designed and made vectors for prokaryotic expression of bomapin, expressed, purified and characterized the protein. TT and GL performed modelling of reduced and oxidized bomapin. MW designed and coordinated experiments, analyzed the data and wrote the manuscript. All authors read and approved the final manuscript.
